# Genetic Divergence among Regions Containing the Vulnerable Great Desert Skink (*Liopholis kintorei*) in the Australian Arid Zone

**DOI:** 10.1371/journal.pone.0128874

**Published:** 2015-06-10

**Authors:** Siobhan Dennison, Steve McAlpin, David G. Chapple, Adam J. Stow

**Affiliations:** 1 Department of Biological Sciences, Macquarie University, North Ryde, NSW, Australia; 2 Australian Wildlife Conservancy, PO Box 8070, Subiaco East, WA, Australia; 3 School of Environmental and Rural Science, University of New England, Armidale, NSW, Australia; 4 School of Biological Sciences, Monash University, Clayton, VIC, Australia; Sichuan University, CHINA

## Abstract

Knowledge of genetic structure and patterns of connectivity is valuable for implementation of effective conservation management. The arid zone of Australia contains a rich biodiversity, however this has come under threat due to activities such as altered fire regimes, grazing and the introduction of feral herbivores and predators. Suitable habitats for many species can be separated by vast distances, and despite an apparent lack of current geographical barriers to dispersal, habitat specialisation, which is exhibited by many desert species, may limit connectivity throughout this expansive region. We characterised the genetic structure and differentiation of the great desert skink (*Liopholis kintorei*), which has a patchy, but widespread distribution in the western region of the Australian arid zone. As a species of cultural importance to local Aboriginal groups and nationally listed as Vulnerable, it is a conservation priority for numerous land managers in central Australia. Analysis of mitochondrial ND4 sequence data and ten nuclear microsatellite loci across six sampling localities through the distribution of *L*. *kintorei* revealed considerable differentiation among sites, with mitochondrial *F_ST_* and microsatellite *F′_ST_* ranging from 0.047-0.938 and 0.257-0.440, respectively. The extent of differentiation suggests three main regions that should be managed separately, in particular the southeastern locality of Uluru. Current genetic delineation of these regions should be maintained if future intervention such as translocation or captive breeding is to be undertaken.

## Introduction

The Australian arid zone occupies 70% of the continent’s landmass and supports an extraordinary biodiversity, including among the world’s richest assemblages of lizards [1], [2]. Despite a longstanding recognition of the conservation value of this region, relatively few studies have described patterns of genetic structuring across whole species distributions [3]. Characterisation of genetic structure across a landscape is valuable to inform conservation because genetically discrete regions may be under different pressures and require separate management approaches. Connectivity among these regions may also not correspond to natural boundaries as expected based on observed environmental or geographic features [4–6]. Moreover, human land use is rapidly changing arid Australia and is posing a number of threats to the biodiversity of the region. Habitat destruction through land clearing, accelerated soil erosion, unsustainable cattle grazing and altered fire regimes continues to threaten inland Australian biota, as do increased weed and feral animal populations [7–9]. Knowledge of levels of genetic connectivity can be used to evaluate the impact of localised activities and to prioritise conservation strategies.

Although the arid regions of Australia currently lack substantial topographic barriers or expansive waterways, genetic variation may be structured by environmental features. Many reptile species are habitat specialists, restricted to specific habitat types scattered throughout the desert region [10,11]. Vast expanses of contiguous habitat types such as dunefields or gibber plain may separate patches of suitable habitat for many species. This may result in habitat patches being isolated from each other by hundreds of kilometres, a distance in excess of the likely dispersal capacity of many desert species [11,12]. Thus, habitat heterogeneity, and the associated habitat specialisation of arid zone lizards, might influence connectivity and restrict gene flow between localities. For example, Chapple et al. [12] found considerable phylogeographic structure within *Egernia inornata* (now *Liopholis inornata* [13]), an arid-Australian lizard, with a number of clades occurring in particular habitat types. It is therefore our *a priori* expectation that localised impacts may threaten genetically distinct components of biodiversity.

There are also additional benefits derived from knowledge of connectivity; for example, it is established that parts of a species distribution that experience prolonged isolation may become sufficiently genetically differentiated that they are worthy of separate management [14–16]. Furthermore, isolation coupled with reduced effective sizes can lower genetic diversity through drift and impinge on the ability to adapt to environmental change. In such cases, translocations among genetically discrete localities may not be a viable conservation strategy owing to the risk of outbreeding depression [16] (but see [17]). Identifying parts of the distribution requiring separate management enables conservation effort to be prioritized and can guide decisions to translocate, restore, or establish breeding programs [16,18].

The great desert skink, *Liopholis kintorei*, is a species endemic to the arid-zone of Australia, currently listed as ‘Vulnerable’ [19]. It is a large scincid lizard that inhabits sand plains, palaeodrainage lines and undulating gravelly downs [20]. Although its range stretches over a vast area of approximately 1.3 million km^2^, it is known to be patchily distributed, with its presence recorded at fewer than 100 localities [21]. Great desert skinks exhibit limited dispersal (commonly 0–4 km, up to 9km; [21]), excavating extensive burrow systems in which close kin live and which may be continuously occupied for up to 7 years [22]. It is a culturally important species to traditional Aboriginal groups [23], and this combined with its threatened status makes its conservation a high priority for land managers in central Australia. Altered fire regimes and the introduction of the red fox and domestic cat are key factors that have led to the species’ decline, as well as habitat decay from feral herbivores [8,19,20,24].

Areas containing great desert skinks are known to occur in a number of geographically distant regions within declared conservation areas: Uluru-Kata-Tjuta National Park (NT), Newhaven Wildlife Sanctuary (NT), Karlamilyi National Park (WA), Ngaanyatjarra Indigenous Protected Area (IPA; WA), and in the Watarru IPA within the Anangu Pitjantjatjara Yankunytjatjara Lands (APY; SA); and in these a number of monitoring and management actions have previously been undertaken. The extent of isolation or genetic differentiation between these regions is unknown, but likely to be high given the apparent disjunct distribution and low dispersal of the species. Here we use mtDNA sequence data (ND4) and ten microsatellite loci to characterise genetic structure and divergence across the range of *L*. *kintorei*. We aim to identify any regions where the genetic distinctiveness of *L*. *kintorei* heightens the conservation value and influences the management options.

## Materials and Methods

### Ethics Statement

All animals were handled in accordance with a protocol considered and approved by Macquarie and Charles Darwin University Animal Ethics committee recommendations (ARA 2008/025 and ARA 2011/037). Sample collection was licensed by the Northern Territory Parks and Wildlife Commission and the South Australian Department of Environment and Heritage.

### Sample collection

**Fig 1:**
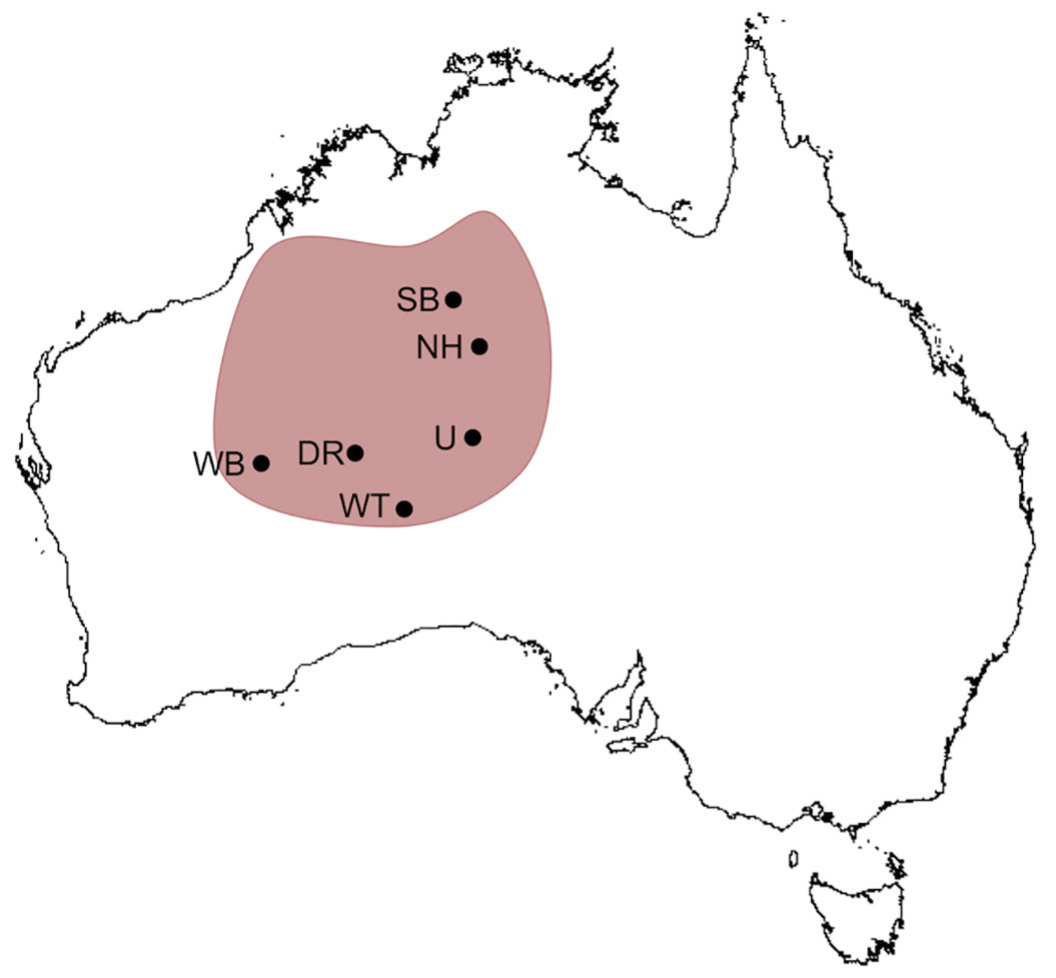
Distribution (shaded) and sampling localities of *L*. *kintorei*. Sangster’s Bore (SB), Newhaven (NH), Uluru (U), Docker River (DR), Watarru (WT) and Warburton (WB). Map derived from data [25] under a CC BY license, with permission from the Commonwealth of Australia, original copyright, 2015.

Ninety-four *L*. *kintorei* samples were collected from six locations throughout the distribution of the species (Fig 1): Australian Wildlife Conservancy’s Newhaven Wildlife Sanctuary (hereafter, Newhaven; 22° 49' 41'' S, 131° 6' 58'' E; *n =* 30) and Sangster’s Bore in the northeast of their distribution (20° 49' 60'' S, 130° 19' 60'' E; *n =* 23), Petalu-Docker River toward the centre of their distribution (hereafter Docker River; 24° 52' 27'' S, 129° 05' 01'' E; *n =* 1), Uluru-Kata-Tjuta National Park to the east (hereafter Uluru; 25° 18' 44'' S, 131° 01' 07'' E; *n =* 30), Warburton to the west within the Ngaanyatjarra IPA (26° 08' 00'' S, 126° 35' 00'' E; *n =* 2) and Watarru within the IPA of the APY Lands at the southern extent of the distribution (27° 11' 43" S, 129° 54' 48" E; *n =* 8). Samples from Warburton were provided by the Western Australian Museum. Tissue was obtained via tail-tip biopsy and preserved in 90% ethanol. *Liopholis kintorei* is a species that exhibits kin-based social living, and high natal philopatry [21]. To ensure that individuals included in our analyses were not highly related, we included only adults captured from separate, distinct burrow clusters.

### Laboratory procedures

Whole genomic DNA was extracted from tissue using a modified salting-out protocol [26]. For each sample the mitochondrial gene, NADH dehydrogenase subunit 4 (ND4), was targeted because previous work on reptiles has shown useful levels of variation for intraspecific studies, though its mutation rate is slow enough to allow inference of deeper divergence due to long-term isolation [12,27]. In addition, individuals were genotyped at ten microsatellite loci to characterise fine-scale population dynamics and more recent divergence.

Polymerase chain reactions (PCR) for all markers were carried out using a PTC-100 Thermocycler (MJ Research, Inc.). Mitochondrial ND4 was amplified using previously developed primers [28]. PCRs were carried out in 20 μL volumes containing 50–100 ng of DNA, 4 uL 5x GoTaq Flexi Buffer (Promega), 2 mM MgCl_2_, 0.2 μM of each dNTP, 0.125 μM of each primer (ND4 and tRNA-leu) and 1 U Taq Polymerase (Promega). Thermocycling began with an initial denaturation for 5 min at 94°C, followed by four touchdown cycles with 94°C denaturation for 30 sec, annealing temperatures (55°C, 53°C, 51°C, 49°C) for 30 sec, and 72°C extension for 45 sec. An additional 35 cycles were carried out at an annealing temperature of 47°C, followed by a final 72°C extension step for 10 min.

Microsatellite PCRs were carried out in 10 μL volumes containing ~50 ng of DNA. A -29 M13 sequence was added to the 5’ end of each forward primer to allow for the incorporation of a complimentary M13 fluorescent-labelled tag, following the protocol of Schuelke [29]. Four tetranucleotide microsatellite loci were developed concurrent to this study (*BX6*, *CKD*, *FQR*, *J3F*; see S1 Text for microsatellite design). In addition to these, we utilised six previously developed markers (*Est1*, *Est2*, *Est9*, *Est12* [30]; *Ecu2*, *Ecu3* [31]). All microsatellite loci were amplified with identical reaction conditions: 2 uL 5x GoTaq Flexi Buffer (Promega), 2.5 mM MgCl_2_, 0.2 μM of each dNTP, 0.02 μM of forward primer, 0.1 μM reverse primer, 0.1 μM of fluoro-labelled tag (FAM, VIC, NED, or PET) and 1 U Taq Polymerase (Promega). Thermocycling began with an initial denaturation for 3 min at 94°C, followed by five touchdown cycles with 94°C denaturation for 30 sec, annealing temperatures (60°C, 58°C, 56°C, 54°C, 52°C) for 30 sec, and 72°C extension for 45 s. An additional 35 cycles were carried out at an annealing temperature of 50°C, followed by a final 72°C extension step for 10 min. PCR products were visualized by electrophoresis on 2% agarose gel. All PCR purification, sequencing and fragment separation was performed by Macrogen (Korea).

### Data analysis

ND4 sequences were checked by eye and aligned with ClustalW, implemented in MEGA 5.0 [32], and submitted to GenBank (Accession numbers KM035773-KM035789). DNA sequences were then translated into amino acid sequences using the vertebrate mitochondrial code. No premature stop codons were observed, indicating that all sequences are true mitochondrial copies. Haplotype and nucleotide diversities were calculated in DnaSP [33].

A minimum-spanning network of ND4 haplotypes was constructed in TCS 1.21 [34]. Global and pairwise *Φ*_*ST*_, an analogue of *F*_*ST*_ [35], were calculated from ND4 haplotypic data in Arlequin v3.5 [36] with 1000 permutations.

Microsatellite alleles were visualised and scored using Peak Scanner 1.0 (Applied Biosystems). To ensure amplification and scoring consistency, at least 10% of samples at each locus were independently rerun and genotyped. Summary statistics, including exact tests for Hardy-Weinberg equilibrium (HWE) and linkage disequilibrium (LD) were conducted in GenAlEx 6.4 [37] and GENEPOP 4.2 [38]. Effective population size (Ne) estimates were calculated utilizing the approximate Bayesian framework implemented in ONeSAMP v1.2 [39]. Due to prohibitively small sample sizes at Docker River and Warburton, these sampling localities were excluded from population-level analyses.

When calculating *F*_*ST*_ analogues from highly polymorphic data such as microsatellites, within-population variance can often approach the level of the total variance, resulting in very low *F*_*ST*_ values even when the populations share no alleles [40,41]. Following Hedrick [40] and Meirmans [41], pairwise fixation index values calculated from microsatellite data (hereafter *F′*_*ST*_) were standardised using the program RECODEDATA 0.1 [41].

STRUCTURE v.2.3 [42] analysis was used to assess genotypic clustering and assignment probabilities. We examined values of *K* = 1–8 (double the number of sample sites included in the analysis), with 10 replicate runs for each, 10^5^ MCMC iterations burn-in and 10^4^ main iterations. Hubisz et al. [43] developed a new model for STRUCTURE, which allows the use of sample-site information. This is different to the initial models including location priors, in that it adds power to analyses, but can disregard site information when true clustering is uncorrelated with sampling locations. We used the ‘admixture’ model with correlated allele frequencies, and repetitions were run with and without location information. The number of genetic clusters (*K*) was determined using the *ΔK* method of Evanno et al. [44].

Discriminant analysis of principal components (DAPC) was used to describe the genetic relationship between sampling localities. DAPC is a multivariate analysis that first uses principal components analysis (PCA) to transform data into uncorrelated components. These components are then analysed using a linear discriminant method, minimising within-group variance while maximising among-group variance [45]. Furthermore, this analysis does not assume HWE and LD, which are often violated when working with natural, small and fragmented populations [45].

DAPC was carried out in the R package adegenet [46], implemented in R 2.12 (R development core team 2013; www.r-project.org), with *K* selected using the find.clusters function and Bayesian Information Criterion (BIC). We also ran DAPC using sample locations as groups (*K =* 4) to assess the differentiation of our sample sites. PCA was performed in R using the dudi.pca function in the package ade4 [47]. Missing data were replaced with the mean (the origin of the X- and Y-axes, as in Horne et al. [48]). Determining the number of principal components (PCs) to retain as predictors for the discriminant analysis requires a balance between the statistical power of more PCs, and the stability of assignments, though there is no strict rule. Retaining too many PCs with respect to sample size can result in over-fitting the data. This trade-off can be assessed using the a.score function in the R package adegenet [46]. Analyses were carried out retaining a conservative 13 PCs, the optimal number suggested by a.score, given our relatively small dataset.

## Results

### Summary statistics

#### Mitochondrial sequence data

**Fig 2:**
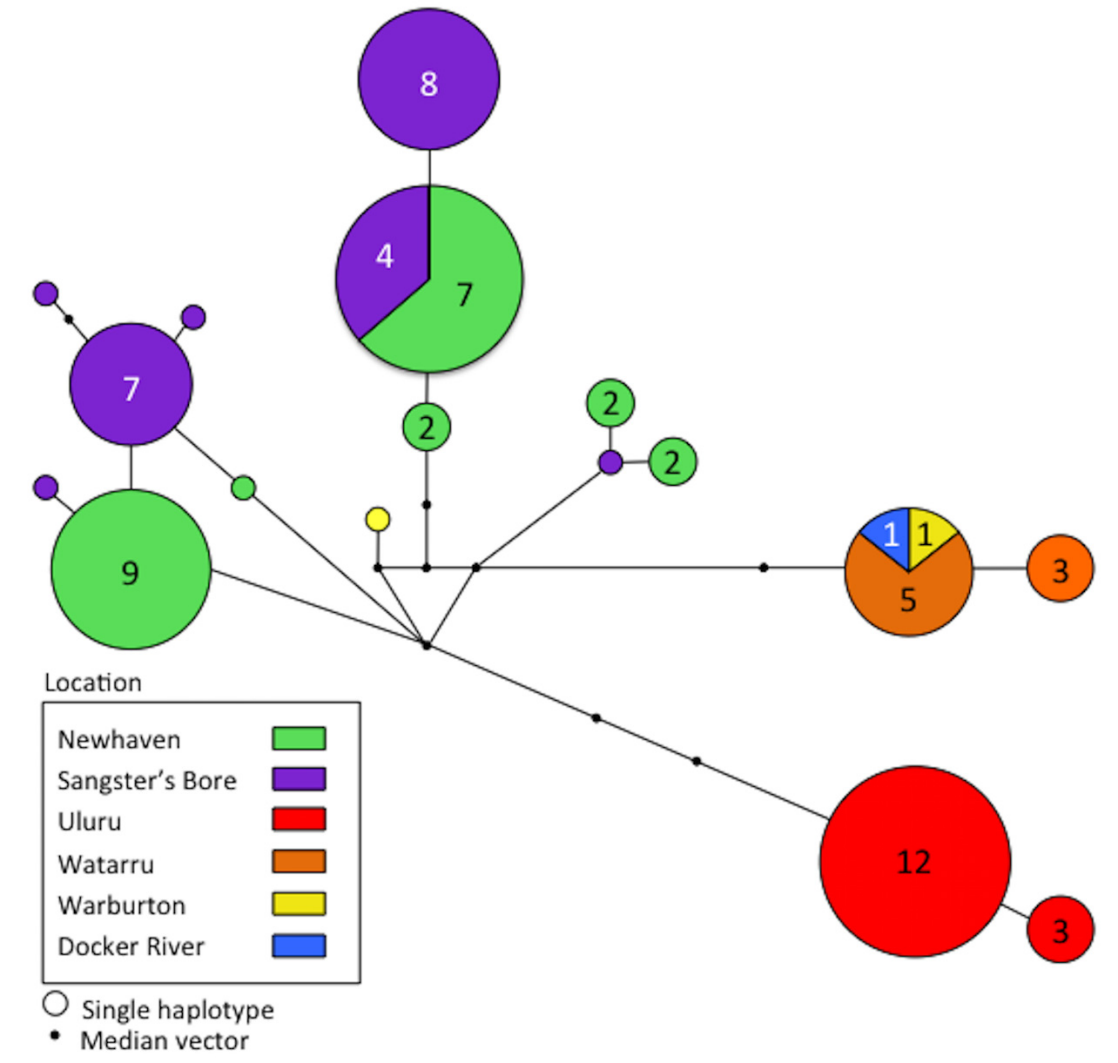
Haplotype network constructed from 72 *Liopholis kintorei* mitochondrial ND4 sequences. Each circle represents a unique haplotype, and the number within indicates its frequency.

Mitochondrial ND4 sequences of 585 bp were successfully amplified from 72 individuals sampled from the six localities. Sequences contained 23 (3.9%) variable sites, of which 18 (3.1%) were parsimony informative, revealing a total of 17 unique haplotypes (Fig 2). Haplotype and nucleotide diversity over all samples were 0.908 and 0.009, respectively, and the variance for both was < 0.0002 (Table 1). All haplotypes sampled at Uluru were unique to that locality. Newhaven and Sangster’s Bore shared haplotypes with only each other, and Docker River and Warburton both shared haplotypes with Watarru. One sample from Warburton was unique to that locality, although small sample sizes may be the reason for this.

**Table 1:** Sample sizes and diversity indices for *Liopholis kintorei* captured from four sampling localities.

Locality	*n* (ND4)	*n*_*h*_	*h*	π	*n* (μsat)	N_a_	R_a_	P_a_	H_O_	H_E_
Newhaven	23	6	0.76	0.0063	30	10.1	6.3	9	0.765	0.780
Sangster’s Bore	23	7	0.78	0.0077	23	10.8	7.2	18	0.811	0.811
Watarru	8	2	0.54	0.0010	8	5.4	5.2	7	0.771	0.688
Uluru	15	2	0.34	0.0006	30	10.5	6.6	18	0.777	0.791
Warburton	2	2			2					
Docker River	1	1			1					

Diversity indices for Warburton and Docker River could not be calculated because of small sample sizes. Number of samples (*n*), number of haplotypes (*n*_*h*_), haplotype diversity (*h*) and nucleotide diversity (*π*) are given for mitochondrial ND4 sequences (total *n* = 72). The number of samples (*n*), average number of alleles per locus (N_a_), allelic richness (R_a_), number of private alleles (P_a_), observed (H_O_) and expected (H_E_) heterozygosities are given over ten microsatellite loci (total *n* = 94).

#### Microsatellite loci

One of the four remaining sample localities was out of HWE (Newhaven, *P < 0*.*05*; Table 2), which may be due to a Wahlund effect from some spatial structure [49]. Following Holm-Bonferroni sequential correction [50], 10 out of 180 locus x locus tests for LD (45 per sampling locality) were significant, all of which were for different locus pairs. The presence of some LD is unsurprising, given that it can be common in threatened species that are expected to have small effective population sizes [18]. Allelic richness of the four sites ranged from 5.2–7.2, and the number of private alleles from 7–18. Overall F_IS_ ranged from 0.053–0.036, with these positive values probably reflecting a spatial Wahlund effect [49]. Ne estimates for each locality were: Newhaven (Ne = 60.4, 95% CI = 38.8–119), Sangster’s Bore (Ne = 41.5, 95% CI = 23.4–116.9), Watarru (Ne = 9.3, 95% CI = 5.8–15.9), Uluru (Ne = 21.2, 95% CI = 16.7–27.6). Estimates of Ne are sensitive to sample size, and as such ours should be treated with caution due to small sample sizes.

**Table 2:** Locus by locus results for Hardy-Weinberg Equilibrium tests for *L*. *kintorei* sample localities.

Locus	Newhaven *(n = 30)*	Sangster’s Bore *(n = 23)*	Watarru *(n = 8)*	Uluru *(n = 30)*
***BX6***	**0.170**	-0.040	-0.140	**0.120**
***CKD***	0.045	0.004	0.143	0.158
***FQR***	-0.019	-0.073	-0.167	0.046
***J3F***	-0.078	0.010	-0.077	-0.169
***EST1***	0.014	**0.130**	-0.217	-0.007
***EST2***	0.036	**0.120**	0.056	**0.192**
***EST9***	-0.063	-0.062	0.167	0.077
***EST12***	**0.217**	-0.081	-0.287	-0.028
***ECU2***	0.017	0.106	-0.021	-0.068
***ECU3***	-0.048	0.047	-0.077	-0.052
***Overall***	**0.036**	**0.025**	**-0.053**	**0.035**

The inbreeding coefficient (F_IS_) for each locus is given, as well as over all ten loci. Bold values denote significant F_IS_ (*P* < 0.05).

#### Genetic differentiation between localities

All comparisons of genetic differentiation, except one (Newhaven-Sangster’s Bore; *P*_*ND4*_ *= 0*.*108*), were high and significant (Table 3), indicating very low connectivity between localities. For ND4, overall *Φ*_*ST*_ = 0.50, *P <* 0.00001. Pairwise population differentiation for both ND4 (*F*_*ST*_) and microsatellites (*F′*_*ST*_) was substantial (Table 3; Juke’s Cantor distances between localities are given in Table 4). Newhaven and Sangster’s Bore were the least differentiated from each other, with low and not-significant *F*_*ST*_ for ND4, though microsatellite differentiation was relatively high. Uluru was the most differentiated from all other localities in all comparisons.

**Table 3:** Pairwise genetic differentiation between four *Liopholis kintorei* sampling localities; generated from 585 bp of the mitochondrial ND4 gene (*F*_*ST*_; lower diagonal) and ten microsatellite loci (*F′*_*ST*_; upper diagonal). All values in bold are significant (*P*_*ND4*_ < 0.0001; *P*_*msat*_ < 0.05).

	Newhaven	Sangster’s Bore	Watarru	Uluru
**Newhaven**		**0.285**	**0.257**	**0.302**
**Sangster’s Bore**	0.047		**0.380**	**0.440**
**Watarru**	**0.494**	**0.502**		**0.263**
**Uluru**	**0.627**	**0.622**	**0.938**	

**Table 4:** Range of Jukes-Cantor genetic distances between four *Liopholis kintorei* sampling localities calculated from ND4.

	Newhaven	Sangster’s Bore	Watarru
**Newhaven**			
**Sangster’s Bore**	0.000–0.016		
**Watarru**	0.007–0.012	0.005–0.014	
**Uluru**	0.007–0.016	0.009–0.017	0.010–0.014

**Fig 3:**
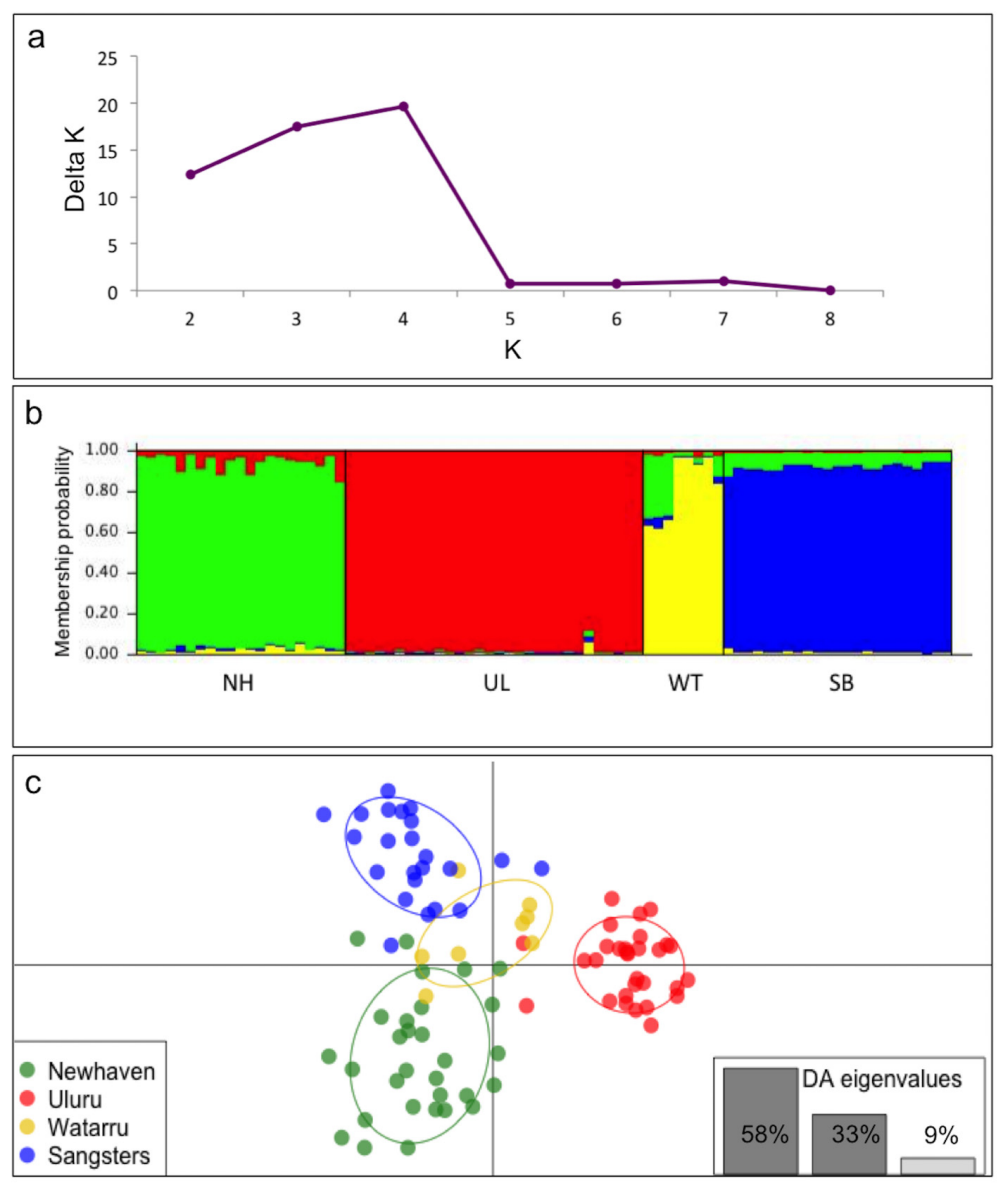
STRUCTURE and DAPC analyses of *Liopholis kintorei* individuals captured from four locations across their distribution: Newhaven (NH), Uluru (UL), Watarru (WT) and Sangster’s Bore (SB). (a) *ΔK* values for each potential number of genetic clusters (*K*) examined, showing a best-fit value of *K* = 4. (b) Bar plot showing population assignment of individuals from each sample locality. (c) DAPC scatter plot showing *K =* 4 genotypic clusters. Each point represents an individual genotype, each sample site being depicted by a different colour and encompassed by a 95% confidence ellipse. Eigenvalue plots represent the amount of genetic variation contained in each discriminant factor.

STRUCTURE analysis yielded a best-fit value of *K =* 2 (S1 Fig) without location information, and *K* = 4 with location information. When *K* = 2 was considered, one cluster comprised the Uluru samples, and the other clumped Newhaven, Sangster’s Bore and Watarru together (S1 Fig). When location information was used, STRUCTURE gave a best-fit value of *K =* 4 (Fig 3a), and there was clear delineation of sample sites with high assignment probabilities (Fig 3b).

A similar result was found in the DAPC: the lowest BIC value was indeed *K =* 2 (BIC = 124) when no location information was used, however the BIC score for *K =* 4 was only slightly higher (BIC = 126; a difference in BIC of 0–2 is considered weak [51]). For *K =* 2, Uluru samples were largely separate from the rest (S1 Fig). However, when samples were grouped by location in the DAPC, there was considerable genetic separation between all four groups, with the largest separation along the first and second discriminant factors lying between Uluru and the other localities (Fig 3c). The optimal number of principal components retained for the DAPC accounted for 54% of the overall variation, and the first two discriminant factors accounted for 91% of this variation in the discriminant analysis. Only plots for *K =* 4 are given here, but see S1 Fig for plots of *K =* 2.

## Discussion

We show genetic partitioning among regions containing the Vulnerable lizard *Liopholis kintorei*. Each of the localities from which *L*. *kintorei* was sampled contained similar levels of genetic variation, and individuals from the Uluru-Kata-Tjuta region were most genetically distinctive (Tables 1, 3 and 4). Our estimates of genetic divergence, in addition to environmental differences experienced among regions, indicate that each of these are reservoirs of important genetic variation and point to the risk of outbreeding depression should interbreeding occur.

Outbreeding depression is lowered reproductive fitness in generations subsequent to crossing of individuals from genetically differentiated parts of their distribution [16]. A decision tree developed by Frankham et al. [16] assists conservation managers to assess the probability of its occurrence, and thus decide whether populations should be kept separate. Applying this to the data for *Liopholis kintorei* indicates a risk of outbreeding depression should individuals be translocated. The third part of the tree suggests that if sites have been isolated from each other for 500 years or more, there is a high risk of outbreeding depression and they should remain separated. A crude estimate of divergence times based on a commonly cited mitochondrial calibration of 1.3–2% sequence divergence per million years [13,52], suggests that the Uluru lineage may have split from the others between 350 kya and 1.31 million years ago. The level of genetic divergence between sampling localities at ND4 (< 2%; Table 4) was below that found in a closely related species, *Liopholis inornata* (within-species divergence up to 6.1% [12]); however, *L*. *inornata* has a much broader distribution and was sampled across a wider area, that may explain the higher within-species divergence reported. When considering clades of *L*. *inornata* sampled across similar geographic scales, the level of differentiation within these two *Liopholis* species was similar.

Furthermore, there are environmental differences between the sites that may contribute to localised adaptation, another factor that flags the possibility of outbreeding depression from translocation according to Frankham et al. [16]. Similarly, Crandall et al. [15] propose evaluating ‘ecological exchangeability’ along with estimates of genetic divergence to decide on the parts of species distributions to be managed separately. Given the latitudinal range over which *L*. *kintorei* is distributed, it is not surprising that there are environmental differences across our sampling localities. For example, mean monthly minimum and maximum temperatures are consistently and significantly cooler at Uluru than the northernmost locality of Sangster’s Bore (see S2 Text for climate data and statistical tests). Average annual rainfall at Sangster’s Bore is 479 mm compared with 320 mm at Uluru (t_32_ = 2.09, *P =* 0.045), though rainfall patterns differ with Uluru receiving more winter rain. Sangster’s Bore experiences more days above 35°C and 40°C than Uluru (respectively, 169.6 vs. 109.3 days ≥ 35°C and 52.9 vs. 32.1 days ≥ 40°C; climate data from Australian Bureau of Meteorology, www.bom.gov.au; data not available for Watarru). Given their ectothermic physiology, life history traits in reptiles have been demonstrated to be linked to altitudinal or latitudinal variability in climate [52–54]. As such, the significant climatic differences between our sampling localities are likely to be relevant to *L*. *kintorei*, and may have led to adaptive differences in, for example, thermal tolerance or seasonal activity.

The habitat in which *L*. *kintorei* is found in these areas varies also. The Sangster’s Bore and Newhaven localities occur along and adjacent to palaeodrainage lines, with the species’ preferred habitat in semi-saline spinifex plains dominated by soft spinifex (*Triodia pungens*) and inland tea tree (*Melaleuca glomerata*). At Watarru, *L*. *kintorei* were found within open mulga woodland or *Eremophila* and woolybutt (*Eragrostis eriopoda)* grass shrubland, and at Uluru they occur on sand plains and flat swales dominated by either hard (*Triodia basedowi*) or soft spinifex (*T*. *pungens* and *T*. *schinzii*) [21].

Uluru was the most genetically distinct in all analyses, and shared no haplotypes with any other site; Watarru was also highly differentiated from the other areas, but shared haplotypes with the other two southwestern localities (Docker River and Warburton). More sampling at Docker River and Warburton is required to determine the true extent of differentiation among these localities. Sangster’s Bore and Newhaven to the north were highly differentiated based on the microsatellite data set (*F′*_*ST*_ = 0.285), but were not significantly differentiated at mitochondrial ND4 (*F*_*ST*_ = 0.047). This discrepancy, taking into account the different inheritance and mutation rates for these genetic markers [55] might indicate a contemporary barrier to dispersal but higher levels of historical gene flow. As a result, we recognise three main delineations among localities for conservation management: one to the north (Sangster’s Bore and Newhaven), one to the southeast (Uluru), and one in the southwest (Watarru, Docker River, Warburton).

Given the isolation of localities implied by an apparently patchy distribution [20,22] and the genetic differentiation among localities investigated here, genetic diversity, if lost, may not be replenished by migration. Effective population sizes are substantially lower than actual sizes in wild populations, with the ratio between them often approximating 0.1 [56], and up to 0.5 [57]. Census size estimates for *L*. *kintorei* are estimated to be low (<500 at Uluru [20]), and genetic Ne estimates in this study were all low (138–232, though these estimates should be treated with caution due to small sample sizes). The Vulnerable status of this species and low estimated population sizes suggest that genetic diversity and viability may be eroded rapidly over time. The threatening processes attributed to the decline of great desert skinks have not been removed, and consequently this is likely to continue. If genetic erosion is allowed to proceed, this can render localised parts of the distribution vulnerable to inbreeding and inbreeding depression [58]. Translocations to bring about a so-called ‘genetic rescue’ have been demonstrated to dramatically reverse the effects of inbreeding depression [17,59]. In the case of *L*. *kintorei*, unique haplotypes at some localities and high differentiation estimates suggest that if this need eventuates, parts of the distribution selected for translocations need to be carefully chosen to avoid the risk of outbreeding depression.

Knowledge of connectivity combined with landscape management of biological processes is needed to conserve biodiversity [15]. Conservation management for *L*. *kintorei* should prioritise the preservation of suitable habitat, in particular addressing recent and localised changes in fire regimes and predation pressure to reduce the risk of further localised population declines and thus erosion of genetic diversity. While further sampling needs to be conducted at Watarru, Docker River and Warburton, the evidence suggests three main delineations for management: (1) Uluru to the southeast, (2) Newhaven and Sangster’s Bore to the north, and (3) Watarru, Docker River and Warburton to the southwest. Uluru in particular should be considered separately for management, and this distinctiveness should be recognised if intervention such as translocation or captive breeding is to be undertaken.

## Supporting Information

S1 Text Characterisation of four polymorphic microsatellite loci for the great desert skink, *Liopholis kintorei*. (DOCX)

S2 Text Climate data and analysis for northern and southern regions containing *Liopholis kintorei*. (DOCX)

S1 Fig STRUCTURE and DAPC analyses of *Liopholis kintorei* individuals using no location information. (a) *ΔK* values for each potential number of genetic clusters (*K*) examined, showing a best-fit value of *K* = 2. (b) Bar plot showing population assignment of individuals from each sample locality. (c) DAPC plot showing *K =* 2 genotypic clusters. The y-axis represents the density of individuals along the given discriminant function. One cluster (red) comprised the Uluru samples, and the other (green) clumped Newhaven, Sangster’s Bore and Watarru together.
(TIFF)
