# Supplementary material for: Genetic Divergence among Regions Containing the Vulnerable Great Desert Skink (Liopholis kintorei) in the Australian Arid Zone
Source: PLoS One. 2015 Jun 10;10(6):e0128874. doi: 10.1371/journal.pone.0128874 (PMC4464518; doi:10.1371/journal.pone.0128874)
Supplement: S1 Text — (DOCX) [file pone.0128874.s001.docx]

Supporting text for:

**Genetic Divergence Among Regions Containing the Vulnerable Great Desert Skink (*Liopholis kintorei*) in the Australian Arid Zone**

S. Dennison, S. McAlpin, D.G. Chapple, A.J. Stow

Correspondence to: siobhan.dennison@mq.edu.au

**Characterisation of four polymorphic microsatellite loci for the great desert skink, *Liopholis kintorei***

Genomic DNA was extracted from tissue samples collected from a closely related species, *Liopholis montana*, using the DNeasy extraction kit (Qiagen) according to the manufacturers protocol. High throughput DNA sequencing was performed with 319ng/µL of DNA on 1/8 of a 70 x 75 PicoTiterPlate using the 454 GS-FLX platform (Roche Applied Science) at the Australian Genome Research Facility. This technique has been described fully elsewhere (Gardner et al. 2011). A total of 207, 381 contigs were sequenced yielding 1,419 microsatellite loci (di-, tri-, tetra-, penta- and hexanucleotide repeats), identified using MSATCOMMANDER version 0.8 (Faircloth 2008). Primer 3 (Untergasser et al. 2012) was used to design primer pairs for 187 loci, following the recommendations of Gardner et al. (2011). Ten of these were selected for amplification trials, four of which successfully cross-amplified in *Liopholis kintorei* and were used in this study (*BX6, CKD, FQR, J3F*).

Polymerase chain reactions (PCRs) were carried out in 10 µL volumes containing ~50 ng of DNA. A -29 M13 sequence was added to the 5’ end of each forward primer to allow for the incorporation of a complimentary M13 fluorescent-labelled tag, following the protocol of Schuelke (2000). Each locus was amplified with identical reaction conditions: 2 uL 5x GoTaq Flexi Buffer (Promega), 2.5 mM MgCl_2_, 0.2 µM of each dNTP, 0.02 µM of forward primer, 0.1 µM reverse primer, 0.1 µM of fluoro-labelled tag (FAM, VIC, NED, or PET) and 1 U Taq Polymerase (Promega). Thermocycling began with an initial denaturation for 3 min at 94 ºC, followed by five touchdown cycles with 94 ºC denaturation for 30 sec, annealing temperatures (60 ºC, 58 ºC, 56 ºC, 54 ºC, 52 ºC) for 30 sec, and 72 ºC extension for 45 s. An additional 35 cycles were carried out at an annealing temperature of 50 ºC, followed by a final 72 ºC extension step for 10 min. PCR products were visualized by electrophoresis on 2% agarose gel. All PCR purification, sequencing and fragment separation was performed by Macrogen (Korea). Microsatellite alleles were visualised and scored using Peak Scanner 1.0 (Applied Biosystems). To ensure amplification and scoring consistency, at least 10% of samples at each locus were independently rerun and genotyped.

Markers were tested on a panel of 55 *Liopholis kintorei* adults captured at the Australian Wildlife Conservancy’s Newhaven Sanctuary, NT, Australia. Tests, including exact tests for Hardy-Weinberg Equilibrium (HWE) and linkage disequilibrium (LD) were conducted in GenAlEx 6.4 (Peakall and Smouse 2006), GENEPOP 4.2 (Raymond and Rousset 1995), with Holm-Bonferroni corrections for multiple tests (Holm, 1979). MICRO-CHECKER 2.2 (van Oosterhout et al. 2004) was used to assess the presence of scoring errors, null alleles and allelic dropout.

Four tetranucleotide microsatellite loci (*BX6, CKD, FQR, J3F*) successfully amplified in *L. kintroei*, with the number of alleles at each locus ranging from 4 - 13. None of the loci showed evidence of scoring errors, null alleles, or allelic dropout. No locus showed any significant deviation from HWE and no pairs of loci were found to be in significant linkage disequilibrium after Holm-Bonferroni correction (Table 1).

**Table S1.1** **Summary statistics and characteristics of four polymorphic microsatellite loci in *Liopholis kintorei*.** Number of samples (*n*), number of alleles per locus (N_a_), observed (H_O_) and expected (H_E_) heterozygosities, and the inbreeding coefficient (F_IS_) are given. None of the loci showed significant deviation from HWE.

| Name | Primer sequence | Motif | Size (bp) | *n* | N_a_ | H_O_ | H_E_ | F_IS_ |
| --- | --- | --- | --- | --- | --- | --- | --- | --- |
| *BX6* | F: TGAGAACCACTGAGCCACAGG  R: ACAGGCAAGTAATAGGCATGAGATAGA | (ATCT)_13_ | 120 – 172 | 55 | 10 | 0.764 | 0.837 | 0.089 |
| *CKD* | F: TGCACCCAATGCACTGACAATGA  R: GACTACAAGCCTCTTAGGAGCAGGA | (CTTT)_15_ | 206 – 254 | 55 | 13 | 0.764 | 0.859 | 0.112 |
| *FQR* | F: TGGTAAATGGCTTTGGGGCCATAC  R: CGAAGTCTAGAGTTGCGAATGGTAGGT | (ACAG)_10_ | 110 – 122 | 55 | 4 | 0.782 | 0.732 | -0.069 |
| *J3F* | F: TGTGATTGGTTTCCCTGATTCAAA  R: TGAGTCAAGATGGAGGACGATGG | (CTGT)­_11_ | 144 – 168 | 55 | 4 | 0.436 | 0.490 | 0.109 |

**References**

Faircloth BC (2008) MSATCOMMANDER: Detection of microsatellite repeat arrays and automated, locus-specific primer design. *Molecular Ecology Resources*, **8:**92–94

Gardner MG, Fitch AJ, Bertozzi T, Lowe AJ (2011) Rise of the machines - recommendations for ecologists when using next generation sequencing for microsatellite development. *Molecular Ecology Resources*, **11:**1093–1101

Holm S (1979) A simple sequentially rejective multiple test procedure. *Scandinavian Journal of Statistics*, **6:**65–70

Peakall R, Smouse PE (2006) GENALEX 6: Genetic analysis in Excel. Population genetic software for teaching and research. *Molecular Ecology Notes* **6:**288–295

Raymond M, Rousset F (1995) GENEPOP (version 1.2): population genetics software for exact tests and ecumenicism. *Journal of Heredity*, **86:**248–249

Schuelke M (2000) An economic method for the fluorescent labeling of PCR fragments. *Nature Biotechnology*, **18:**233–234

Untergasser A, Cutcutache I, Koressaar T, Ye J, Faircloth BC, Remm M, Rozen SG (2012) Primer3-new capabilities and interfaces. *Nucleic Acids Research*, **40:**e115

Van Oosterhout C, Hutchinson WF, Wills DPM, Shipley P (2004) MICRO-CHECKER: software for identifying and correcting genotyping errors in microsatellite data. *Molecular Ecology Notes*, **4:**535-538
