# Supplementary material for: Genetic Divergence among Regions Containing the Vulnerable Great Desert Skink (Liopholis kintorei) in the Australian Arid Zone
Source: PLoS One. 2015 Jun 10;10(6):e0128874. doi: 10.1371/journal.pone.0128874 (PMC4464518; doi:10.1371/journal.pone.0128874)
Supplement: S2 Text — (DOCX) [file pone.0128874.s002.docx]

Climate data from the Australian Bureau of Meteorology (http://www.bom.com.au)

**Table S2.1** Monthly average maximum temperature (T_max_), average minimum (T_min_) temperature and average rainfall for Sangster’s Bore (station no. 015666, Rabbit Flat), Northern Territory. Data shown are averaged over the last 17 years of data (1997-2013), and standard errors are given in parentheses

|  | JAN | FEB | MAR | APR | MAY | JUN | JUL | AUG | SEP | OCT | NOV | DEC | ANNUAL |
| --- | --- | --- | --- | --- | --- | --- | --- | --- | --- | --- | --- | --- | --- |
| Mean T_max_ | 38.81 (0.44) | 37.28  (0.47) | 35.96  (0.58) | 33.92  (0.46) | 28.24  (0.81) | 25.46  (0.35) | 26.08  (0.28) | 29.54  (0.38) | 34.49  (0.39) | 37.14  (0.44) | 38.51  (0.34) | 38.36  (0.40) | 33.65  (0.24) |
| Mean T_min_ | 24.08  (0.28) | 23.48  (0.20) | 21.72  (0.36) | 16.74  (0.49) | 11.74  (0.52) | 7.68  (0.56) | 6.86  (0.65) | 8.43  (0.44) | 14.51  (0.51) | 18.47  (0.45) | 21.95  (0.33) | 23.52  (0.16) | 16.59  (0.24) |
| Rainfall | 105.05  (22.24) | 98.14  (19.38) | 79.26  (28.02) | 22.43  (8.72) | 14.60  (5.62) | 7.34  (2.85) | 4.78  (2.79) | 1.42  (0.98) | 6.18  (3.01) | 21.37  (5.08) | 42.69  (8.47) | 76.15  (11.16) | 479.41  (58.89) |
| Days ≥ 35 ºC^✝^ | 25.9 | 20.6 | 21.0 | 11.3 | 0.9 | 0.0 | 0.0 | 1.7 | 15.9 | 23.5 | 24.4 | 24.4 | 169.6 |
| Days ≥ 40 ºC^✝^ | 12.6 | 7.3 | 2.7 | 0.7 | 0.0 | 0.0 | 0.0 | 0.0 | 0.6 | 7.6 | 11.3 | 10.1 | 52.9 |

^✝^ *standard errors could not be calculated, as data for individual years were not available for these variables. Data averaged from 1996-2013.*

**Table S2.2** Monthly average maximum temperature (T_max_), average minimum (T_min_) temperature and average rainfall for Uluru (station no. 015635, Yulara Airport), Northern Territory. Data shown are averaged over the last 17 years of data (1997-2013), and standard errors are given in parentheses

|  | JAN | | FEB | | MAR | | APR | | | MAY | JUN | | | JUL | | AUG | SEP | | OCT | NOV | | | DEC | | ANNUAL | |
| --- | --- | --- | --- | --- | --- | --- | --- | --- | --- | --- | --- | --- | --- | --- | --- | --- | --- | --- | --- | --- | --- | --- | --- | --- | --- | --- |
| Mean T_max_ | 38.68  (0.39) | | 36.65  (0.50) | | 33.62  (0.51) | | 29.83  (0.51) | | | 24.36  (0.50) | 20.09  (0.28) | | | 20.35  (0.41) | | 23.82  (0.46) | 29.29  (0.53) | | 32.15  (0.56) | 34.35  (0.51) | | | 35.95  (0.39) | | 29.94  (0.22) | |
| Mean T_min_ | 23.22  (0.39) | | 22.53  (0.39) | | 19.35  (0.49) | | 14.65  (0.37) | | | 9.26  (0.51) | 5.21  (0.45) | | | 4.54  (0.41) | | 6.12  (0.34) | 11.09  (0.33) | | 15.04  (0.41) | 18.46  (0.35) | | | 20.97  (0.30) | | 14.20  (0.20) | |
| Rainfall | 28.22  (5.23) | | 51.21  (20.70) | | 40.69  (13.60) | | 15.07  (6.55) | | | 10.34  (4.66) | 20.95  (9.21) | | | 19.53  (6.77) | | 5.45  (1.75) | 8.27  (4.19) | | 26.35  (8.80) | 47.81  (7.53) | | | 46.11  (12.67) | | 320.01  (48.57) | |
| Days ≥ 35 ºC^✝^ | | 25.3 | | 19.3 | | 14.3 | | 2.9 | 0.1 | | | 0.0 | 0.0 | | 0.0 | | 3.4 | 9.8 | | | 14.3 | 19.9 | | 109.3 | |  |
| Days ≥ 40 ºC^✝^ | | 11.8 | | 6.9 | | 1.7 | | 0.0 | 0.0 | | | 0.0 | 0.0 | | 0.0 | | 0.0 | 0.8 | | | 4.2 | 6.7 | | 32.1 | |  |

^✝^ *standard errors could not be calculated, as data for individual years were not available for these variables. Data averaged from 1983-2013.*

**Table S2.3** Results of independent t-tests comparing average maximum temperature (T_max_), average minimum (T_min_) temperature and average rainfall at Sangster’s Bore and Uluru, Northern Territory from 1997-2013. Results are given for the hottest month (January), the coldest month (July), and the annual average

| Variable |  | *n* | df | t | *P-*value |
| --- | --- | --- | --- | --- | --- |
| T_max_ | January | 17 | 32 | 0.22 | 0.414 |
|  | July | 17 | 32 | 11.43 | <0.001 |
|  | Annual | 17 | 32 | 11.36 | <0.001 |
| T_min_ | January | 17 | 32 | 1.80 | 0.040 |
|  | July | 17 | 32 | 3.01 | 0.003 |
|  | Annual | 17 | 32 | 7.54 | <0.001 |
| Rainfall | January | 17 | 32 | 3.36 | 0.001 |
|  | July | 17 | 32 | -2.01 | 0.026 |
|  | Annual | 17 | 32 | 2.09 | 0.045 |
