# Supplementary figures and images for: Genetic Divergence among Regions Containing the Vulnerable Great Desert Skink (Liopholis kintorei) in the Australian Arid Zone
Source: PLoS One. 2015 Jun 10;10(6):e0128874. doi: 10.1371/journal.pone.0128874 (PMC4464518; doi:10.1371/journal.pone.0128874)

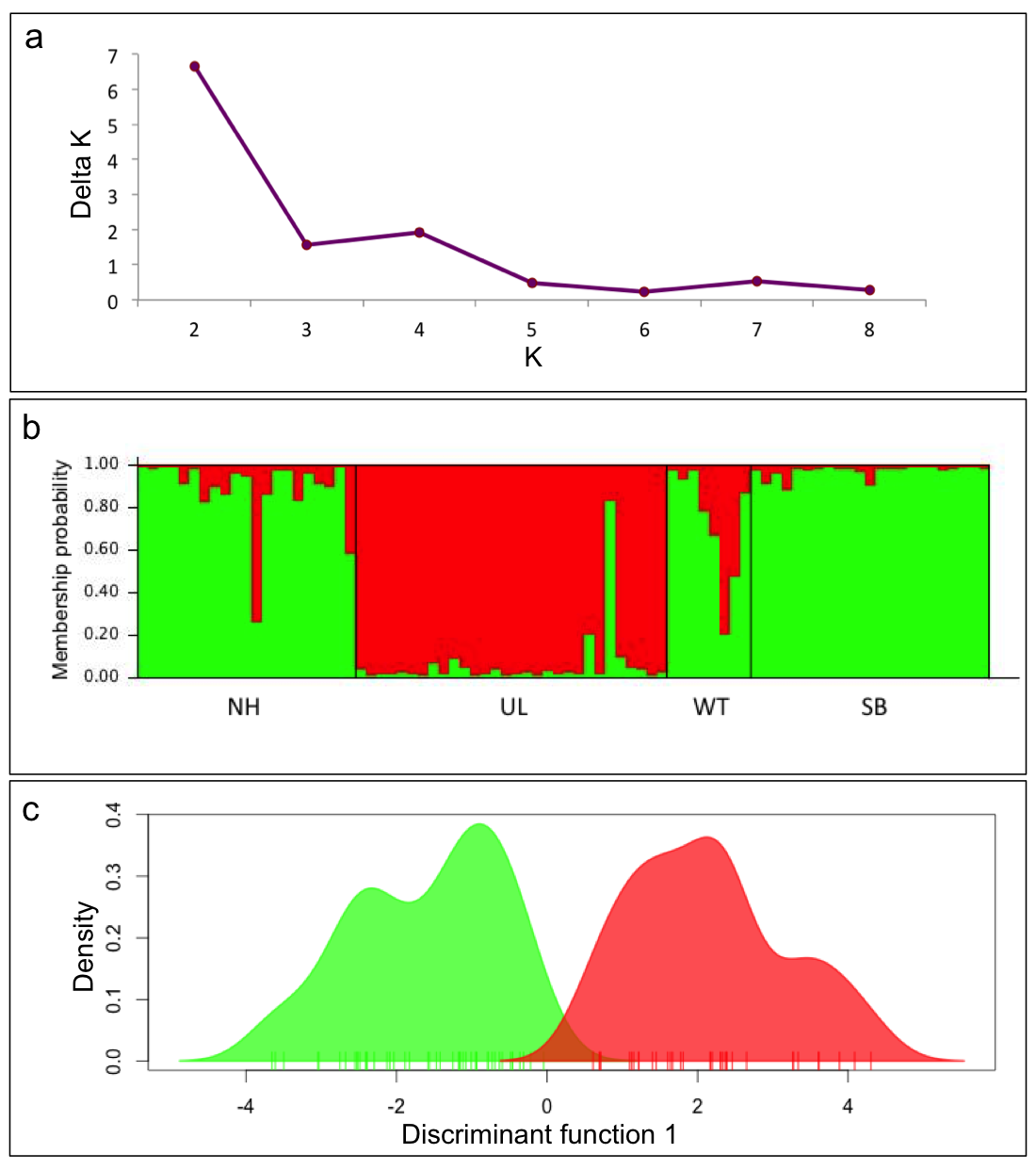

Supplement: S1 Fig — (a) ΔK values for each potential number of genetic clusters (K) examined, showing a best-fit value of K = 2. (b) Bar plot showing population assignment of individuals from each sample locality. (c) DAPC plot showing K = 2 genotypic clusters. The y-axis represents the density of individuals along the given discriminant function. One cluster (red) comprised the Uluru samples, and the other (green) clumped Newhaven, Sangster’s Bore and Watarru together. (TIFF) [file pone.0128874.s003.tiff]
